# Situational analysis of malaria in Cabo Verde: From endemic control to elimination, history, cases data and challenges ahead

**DOI:** 10.1371/journal.pgph.0004153

**Published:** 2025-01-10

**Authors:** Adilson José DePina, Jonas António Lopes Gomes, António Lima Moreira, El Hadji Amadou Niang

**Affiliations:** 1 Programa de Eliminação do Paludismo, CCS-SIDA, Ministério da Saúde, Praia, Cabo Verde; 2 Instituto Nacional de Saúde Pública, Praia, Cabo Verde; 3 Programa Nacional de Luta contra as doenças de transmissão Vectorial e Problemas Ambientais, Ministério da Saúde, Praia, Cabo Verde; 4 Laboratoire d’Ecologie Vectorielle et Parasitaire (LEVP), Université Cheikh Anta Diop de Dakar, Dakar, Senegal; University of Oxford, UNITED KINGDOM OF GREAT BRITAIN AND NORTHERN IRELAND

## Abstract

On 12 January 2024, Cabo Verde was officially certified by the WHO as a malaria-free country after six consecutive years without local transmission. This study analysed the malaria history of Cabo Verde from 1953 to certification in 2024, highlighted the valuable lessons learned, and discussed challenges for prevention reintroduction. Malaria data from the last 35 years (1988–2022) were analysed using descriptive analyses, and cases were mapped using the USGS National Map Viewer. From 1988 to 2022, 3,089 malaria cases were reported, 2.381 (77.1%) locally and 708 (22.9%) imported. Imported cases were reported nationwide except on Brava Island. Six municipalities did not report any cases, while local cases were restricted to Santiago and Boavista, with 2.360 and 21 cases, respectively. Malaria history in the country revealed six remarkable steps and three periods of interruption in the transmission of local malaria cases. The last local cases were reported in Boavista in 2015 and Santiago in 2017. Since 2018, introduced cases have been recorded from time to time. Disease lethality was low, with ten malaria deaths from 2010 to 2023, and the highest value of 8.3% (3/36) recorded in 2011. With this certification, Cabo Verde became a reference in Africa for its health sector organisation, multisectoral, and partnership in malaria control. However, maintaining the certification presents several sustainability challenges for the country. Additionally, robust epidemiological and entomological surveillance, continued investigations, and ongoing research are crucial.

## Introduction

In 2016, the World Health Organization (WHO), through the Initiative E-2020, identified 21 countries in the five regions that could defeat malaria by 2020, considering the likelihood of elimination across critical criteria. All were united by one goal: to achieve zero Indigenous cases within the 2020 deadline [1].

According to the World Malaria Report 2023, there are approximately 249 million malaria cases and an estimated 608,000 malaria deaths, compared to 244 million cases and 610,000 deaths in 2021. Africa continues to carry a disproportionately high share of the global malaria burden, with approximately 94% of all malaria cases and 95% of deaths, and children under five years of age, with approximately 78% of all malaria deaths. Four African countries were responsible for more than half of all malaria deaths worldwide, namely Nigeria (26.8%), the Democratic Republic of the Congo (12.3%), Uganda (5.1%) and Mozambique (4.2%) [2].

Despite the high numbers in Africa, there have been significant gains in reducing and eliminating cases in certain countries. According to the WHO, around a hundred countries and territories worldwide have achieved at least three consecutive years without indigenous malaria cases and have been certified as a “malaria-free country” [3]. Kyrgyzstan received elimination certification in 2016 [4], and Paraguay and Uzbekistan were certified in 2018 [5,6]. In 2019, two other countries obtained certification, notably Algeria in North Africa and Argentina on the American continent [7]. In 2021, WHO certified China as malaria-free, and El Salvador became the first country in Central America to achieve this goal [8]. In 2023, three other countries were certified: Azerbaijan, Tajikistan, and Belize [3].

Cabo Verde, a member of Initiative E-2020, has been recording malaria cases since the 16^th^ century, during the colonisation of the islands by migrants from Africa (including North Africa), Spain, Italy, and Portugal [9]. The disease was endemic and attenuated by certain epidemics, particularly in high rainfall or a considerable influx of migrants from endemic regions, notably São Tomé and Principe, Angola, and Guinea-Bissau [10]. The last indigenous case in the country was diagnosed in January 2018. After three years of accomplishing this great goal, in 2022, the government requested certification as a malaria-free country by the WHO. Cabo Verde was declared malaria-free on 12 January 2024 and became the first malaria-free country in sub-Saharan Africa.

This historic gain in Africa, a continent endemic to malaria, means that Cabo Verde is the fourth country certified after Mauritius, Morocco, and Algeria, certified in 1973, 2010, and 2019, respectively [3]. The country will now face enormous challenges in maintaining zero local cases, perpetuating gains and sustainability, and maintaining a robust and functional surveillance system.

This study provides an overview of the central historical moments of malaria in Cabo Verde, from control to certification. It presents data from the last 35 years of malaria in the country (1988–2022), the distribution of these cases by islands and municipalities, the instituted control measures, lessons learnt, and some challenges for the future prevention of malaria re-introduction in Cabo Verde.

## Methodology

### Description of the country

**Fig 1:**
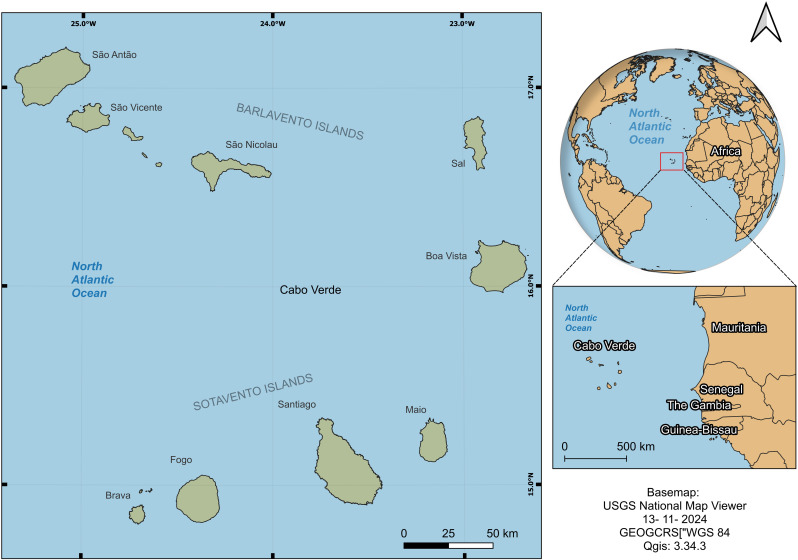
Localisation of Cabo Verde Islands. The map was created using the USGS National Map Viewer (https://www.usgs.gov/tools/national-map-viewer).

Cabo Verde is a volcanic archipelago located approximately 450 km from the West African coast, west of Dakar, Senegal. It occupies an area of 4033 km² and comprises ten islands, nine inhabited, and several islets (Fig 1). Depending on their position relative to the prevailing northeast wind, the islands are divided into two groups: Barlavento to the north and Sotavento to the south, depending on where the wind blows and where the wind flows.

The islands are relatively dispersed, and the terrain is very rugged, with only approximately 10% of their land suitable for agriculture. The country has 22 municipalities and 24 cities, each with a set of health infrastructures that provide various services at different levels of healthcare. According to the latest population census in 2021 and the 2010–2040 projection of the National Institute of Statistics [11], the archipelago had 509,078 residents in 2023. Among these, 67.9% of households and 64 3% of the population live in urban areas. Santiago Island is the largest in the country and is home to more than half of the population (56.7%), followed by Praia (27.5%) and São Vicente (14.7%). Regarding the age structure, the country is considered young, with 29% of residents aged 0–14, 19.2% aged 15–24, and 46.3% aged 25–64. People 65 or over comprise 5.5% of the population [11].

Surrounded by the sea, Cabo Verde is an archipelagic country characterised by a temperate climate and stable temperatures with extreme aridity. Average annual temperatures are generally moderate, around 25 °C, due to maritime influence, with the highest monthly average temperatures in September (warmest season, 26.7 °C) and lowest during the cold season (January/February, 18.4 °C) [12]. The average annual relative humidity of the air varies from 75% in arid lowlands to more than 80% in the highest-lying areas. Depending on the terrain, climate, and vegetation type, the following bioclimatic zones are considered: i) Arid coastal zone, with altitudes of 0 to 200 m, annual rainfall less than 300 mm, where the vegetation is generally steppe-type herbaceous; ii) semi-arid zone, at 200–400 m altitude, with an annual rainfall of 300–400 mm and natural vegetation, although more diverse, differs little from that of the arid coastal zone; iii) Subhumid zone, at 400–600 m altitude, with precipitation of 400–600 m; this area is instead occupied by agriculture and there are several shrubs and tree species there; and iv) wetland, above 700 m, with an average rainfall above 600 mm, particularly on north-facing slopes.

### Literature review

An analysis of published documents on malaria in Cabo Verde was carried out using various sources, namely Google Scholar, PubMed, and WHO. In addition, available but unpublished documents were used in this process, namely documents and guidelines from the Ministry of Health (MS in Portuguese) and the National Malaria Control Program (NMCP) in Portuguese, French, or English. This literature review also used epidemiological, programmatic, demographic, and social data. Due to the limited data sources, no formal exclusion criteria were instituted.

### Malaria data collection and analyses, 1988–2022

According to national guidelines, all malaria cases diagnosed in health facilities (hospitals, health centres, health posts, or private services) are immediately communicated to the Health Delegation. In turn, the Health Delegation immediately reports cases to the central level. The NMCP and the Integrated Surveillance and Epidemic Response (SVIRE, in Portuguese) then compile the data. All cases were classified according to WHO guidelines as imported if the infection was acquired outside the country or indigenous if acquired locally without evidence of importation.

Once a case is confirmed and reported, the Health Delegation responsible initiates a reactive investigation. This consists of visiting the patient’s home, performing reactive rapid diagnostic tests (RDTs) in neighbouring houses, focal spraying activities, community sensitisation, and collecting entomological data.

The data used in this study included malaria data collected from all health structures at the national level during the study period. All data were confirmed between the NMCP and SVIRE services to ensure uniformity and quality of the disclosed data, usually reported through the Ministry of Health’s annual statistical reports.

This study analysed data collected over 35 years, from 1988 to 2022, using simple analysis in Excel and creating case distribution maps. For map elaboration, the shapefile layers with administrative limits defined by the National Institute of Territory Management (INGT, https://ingt.gov.cv/ingt/) and USGS National Map Viewer (https://www.usgs.gov/tools/national-map-viewer). The data were disaggregated into 5-time serials of seven years each, considering the number of years with available data, multiples of seven. The distribution was analysed by the origin of the case, classified as local or imported, according to the WHO guidelines, island, and reporting municipality.

### Mapping the data

We used the USGS National Map Viewer, to prepare malaria maps. First, we obtained georeferenced malaria data from the Cabo Verde NMCP. We then created a variable to join the Shapefile to the Excel base and imported the data into USGS National Map Viewer, using the union tool. After importing, we represented malaria cases using gradual colours, depending on each municipality’s cumulative frequency of cases. We use shapefile layers with administrative limits defined by the INGT. We used the USGS National Map Viewer print composer to finalise and export the maps in the PNG format at 600 DPI. The maps contained a legend to make it easier for users to interpret the data.

## Results

### Epidemiological history of malaria in Cabo Verde

Historical data suggest that malaria was introduced to Cabo Verde by migrants from Africa during the colonisation of the islands. Preliminary data indicate that in 1506, Indian route caravels were banned from Cabo Verde (Santiago) owing to malaria [10]. At that time, the disease was listed as relatively attenuated endemic, with epidemics of some magnitude, particularly in years of high rainfall or increasing migration of individuals from highly endemic regions such as S. Tomé and Príncipe, Angola, or Guinea-Bissau [13–15].

Malaria severely affected the archipelago during the first half of the 20^th^ century. Between 1930 and 1940, it accounted for more than half of hospitalisations, with more than 10,000 cases and 200 deaths per year, particularly in the islands of S. Vicente, Sal, Maio, Boavista and Santiago. Specifically, in 1931, it was responsible for 51.4% of hospitalisations, 36.6% in 1938, and 55.6% in 1940. By the early 1950s, it was the leading cause of mortality, with meso-endemic transmission occurring in Santiago, Fogo, Boavista, São Vicente, and São Nicolau. On the other hand, four other islands were spared, namely Maio, Brava, Sal, and Santo Antão. At that time, the disease was characterised by its marked seasonality, with an annual incidence of > 100 cases per 1,000 inhabitants [15,16].

By analysing the malaria data in the country, it is possible to highlight six reference stages in the fight against malaria, emphasising three periods of interruption in the transmission of the disease. The first period started in the 1940s and continued until 1972, when malaria constituted a severe public health problem, with significant epidemics leading to a situation of hyperendemicity [17]. To combat the disease in the country, a pilot elimination project was launched on the island of Sal in 1948. Because of the good results obtained, it was extended to other islands and municipalities in 1953. The main activities during Indoor residual spraying (IRS) campaigns include the use of dichlorodiphenyltrichloroethane (DDT) against resting adult mosquitoes and a vigorous campaign against larvae, using larvicides, diesel and larvivorous fish (*Gambusie affinis*) in affected areas. With this campaign, it was possible to eliminate the disease from the island of Sal (in 1950), S. Vicente (in 1954), Boavista and Maio (in 1962) and Santiago (in 1968), and the last case was recorded in Santiago in 1967 [17].

Cabo Verde aligned with the WHO global program for eliminating malaria through the biannual IRS campaigns conducted in all islands. It was possible to interrupt malaria transmission twice, from 1972 to 1985. This first phase of interruption of transmission lasted five years; then, in 1973, there were 149 cases of indigenous people in Santa Catarina and Santa Cruz on the island of Santiago. Implementing preventive measures, mainly the treatment of active outbreaks in 1974 and 1975, reduced the number of cases from 149 in 1973 to 20 indigenous cases in 1976. However, a new epidemic from 1977 to 1979, with 844 cases and 13 deaths, peaked in 1978 and again in the municipalities of Santa Catarina and Santa Cruz. In 1978, the epidemic peaked in November, and one-third of the indigenous cases were due to *P. vivax*. New preventive measures have also been implemented. IRS campaigns were conducted every six months for five years, from 1978 to 1983, resulting in an interruption of transmission for three years, from 1983 to 1985 [17]. In 1987, another outbreak led to a declaration of 434 cases. In 1988, a total of 814 cases and 12 deaths were reported, mainly concentrated on Santiago Island. Since the 1990s, imported cases have been diagnosed throughout the country, with indigenous cases limited to the island of Santiago and occasional cases of Boavista since 2003. Since 1990, the transmission level in Cabo Verde has remained very low, and malaria has ceased to be a significant public health problem [17]. Between 1990 and 2009, 1,293 cases were reported nationally - 94% in Santiago. Of the reported cases, 915 were indigenous to the island of Santiago and 14 on Boavista. Since 1996, São Nicolau and Maio have not reported any cases, while five other islands have reported only 45 imported cases. In 2003, four autochthonous cases were reported in Boavista, among residents with no history of travel outside the country during the expected period of contracting the infection. These cases confirmed the presence of active malaria transmission outside Santiago Island for the first time in the past 30 years.

**Fig 2:**
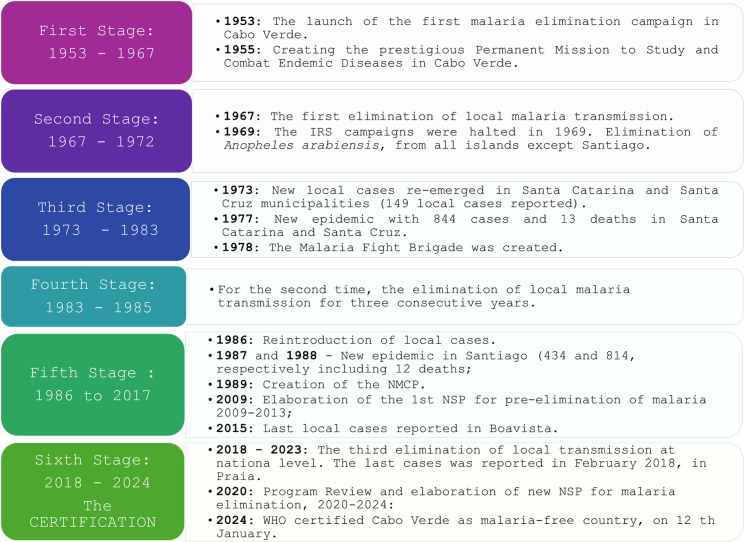
Central moments in malaria elimination history in Cabo Verde. From the first moments of the fight against the disease (1953) to eliminating and certifying the country as malaria-free (2024).

The third period is between 2018 and 2024, when the country established its elimination goal and elaborated on all documents and strategies to achieve it. The interruption of local cases from 2018 to 2024 lasted for five years. The Fig 2 shows the principal historical moments of malaria interventions in Cabo Verde, the results and the gains regarding malaria control and the main activities implemented in Cabo Verde, from the first moments of the fight against the disease in 1953 to eliminating and certifying the country as malaria-free in 2024 (S1 Table)

### Malaria data in Cabo Verde from 1988 to 2022

**Fig 3:**
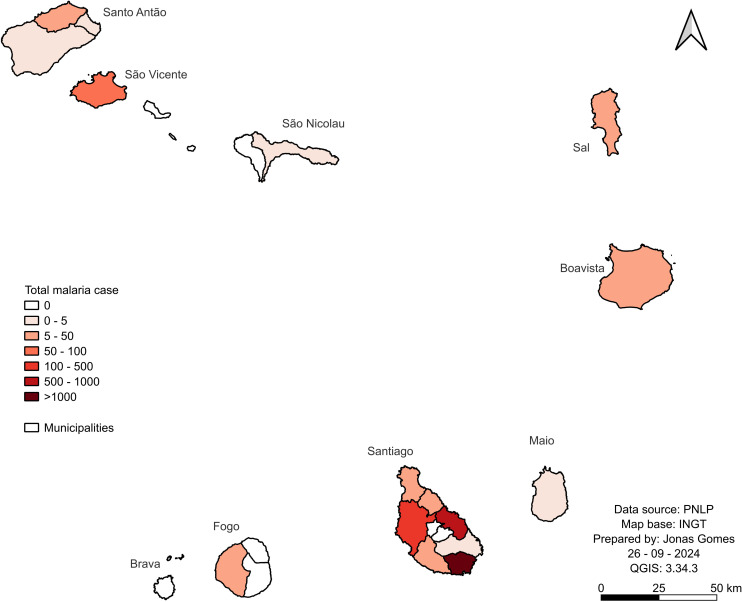
Total malaria cases in the Cabo Verde Islands from 1988 to 2022. The map was created using the USGS National Map Viewer (https://www.usgs.gov/tools/national-map-viewer).

For 35 years, 3,089 malaria cases were reported, 2,381 (77.1%) local and 708 (22.9%) imported (Fig 3 and S2 Table). The first period, 1988–1994, had the highest number of cases, 1,199. Of these, 1,068 (89.1%) were local, and 131 (10.9%) were imported). In the second period, 1995–2001, 606 cases were reported, 484 (79.9%) local and 122 (20.1%) imported. The third period, 2002–2008, has the lowest number of cases, 343, 230 (67.1%) were local and 113 (32.9%) imported. The fourth period, 2009–2015, had 302 cases reported and was the only period where the number of local cases, 125 (41.4%), was fewer than the imported, 177 cases (58.65). And finally, in the fifth period, 2016–2022, there were 639 cases, 474 (74.2%) local and 165 (25.8%) imported.

Most cases were registered on the island of Santiago, with 2911 (94,2%) of the country’s largest island, comprising nine municipalities. The highest cases were in Praia, 1,453 (47.0%), the country’s capital, followed by Santa Cruz, 926 (30%), Santa Catarina, 471 (15.2%) and Tarrafal, 43 (1.4%). Boavista had 37 cases (1.2%), 21 local and 16 imported, and the island of Sal had 34 (1.1%), all imported. Fogo (municipality of São Filipe), Santo Antão (Ribeira Grande and Paúl), as well as the island of Maio, São Nicolau (Ribeira Brava), reported less than 20 cases each (with 17, 7, 4, 3 and 1 cases respectively). Tarrafal in São Nicolau, Brava, Santa Catarina, and Mosteiros in Fogo, São Salvador do Mundo, and São Lourenço dos Órgãos in Santiago have not reported cases during this period of 35 years.

### Imported cases by islands/municipalities

**Fig 4:**
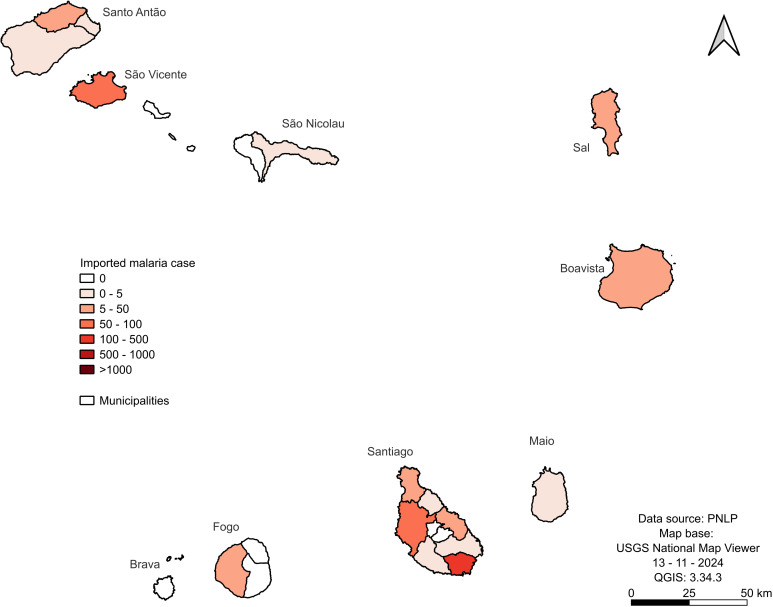
Total of imported malaria cases by the Cabo Verde Islands from 1988 to 2022. The map was created using USGS National Map Viewer (https://www.usgs.gov/tools/national-map-viewer).

Despite the imported cases being inferior to the local ones, they had greater dispersion across islands. Santiago is still the island with prominent imported cases, with a total number of 551 cases, distributed by the 7/9 municipalities, being in Praia (447), Santa Catarina (59) Santa Cruz (22), Tarrafal (29), São Miguel (4), São Domingos (3) and Ribeira Grande (2) (Fig 4), São Lourenço dos Órgãos and São Salvador do Mundo are the two municipalities reporting 0 cases during the period.

Santo Antão reported imported cases in all three municipalities: seven in Ribeira Grande, four in Paul, and two in Porto Novo. São Vicente is the second island in terms of imported cases (73) after Santiago. In São Nicolau, only three malaria cases have been reported in Ribeira Brava and Tarrafal without any case. Sal, Boavista, and Maio Islands reported 34, 16, and 1 case, respectively. In Fogo, only São Filipe reported cases (17), while Santa Catarina and Mosteiros reported no cases. And Brava, reported zero cases during the period.

### Local malaria cases by islands/municipalities

**Fig 5:**
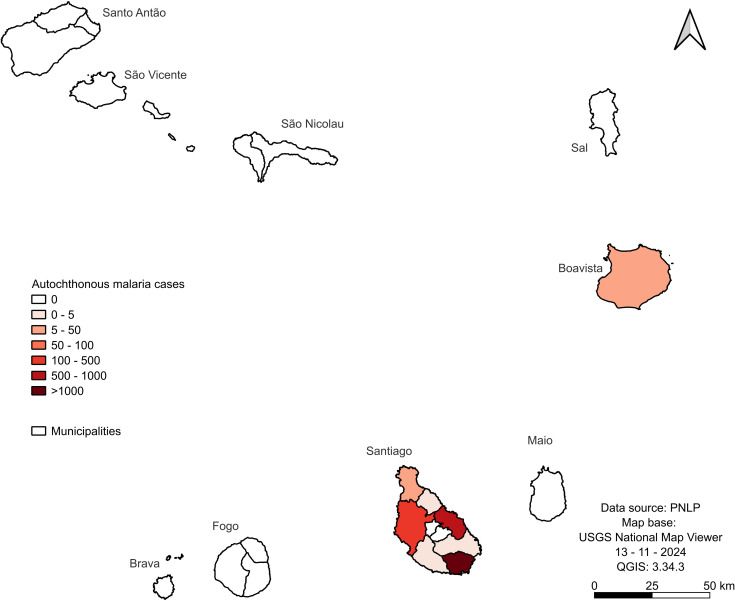
Total local malaria cases in the Cabo Verde Islands from 1988 to 2022. The map was created using USGS National Map Viewer (https://www.usgs.gov/tools/national-map-viewer).

The local malaria cases during this period were restricted to the sole island of Santiago, in Praia (1,006), Santa Cruz (904), Santa Catarina (412), Tarrafal (29), São Miguel and Ribeira Grande (4 cases each), São Domingos (1 case), and Boavista (21 cases) (Fig 5). The other seven islands did not report local cases during this period. Since January 2018, no local cases have been reported in the country; however, six introduced instances have been identified in Praia.

### The dynamics of malaria transmission and level of endemicity

Malaria in Cabo Verde was previously endemic, with an annual incidence of approximately 100 cases per 1,000 inhabitants in the 1950s. With the eradication campaigns launched in Sal, then extended to all the islands, disease transmission stagnated throughout the country, except the island of Santiago [18]. A few years later, the disease resurfaced in Santiago, where major epidemics were recorded and were limited to the island, which remained until 2003 as the only one with indigenous cases. Indigenous cases have also appeared on Boavista island [17–19].

In general, it is believed that the entire Cape Verdean population is at a risk of contracting malaria. Based on the established epidemiological classification, the presence of vectors and the reporting of indigenous cases, with the risks being high, intermediate, or low. Thus, the risk is high on islands with vector and local transmission, where 58% of the population lives. Islands with vectors without local transmission account for 37% of the population and are classified as intermediate risk. Finally, islands without vectors and local transmission where 5% of the population live are classified as low risk [20]. In addition to the low immunity of the population, the dynamics of the internal mobility of the country and the movements of populations from other endemic countries, among other factors, mean that the entire population is considered at risk of malaria transmission.

### Malaria morbidity and mortality in Cabo Verde

The data indicate that from 1989 to 1994, the number of malaria cases decreased: 284 cases and only 2 cases in 1994. From 1992 to 1994, all indigenous cases were on the outskirts of Praia. After heavy seasonal rains and periods of drought, the number of cases increased, with outbreaks in Santa Catarina, Praia, and Santa Cruz, all on Santiago Island. Faced with this morbidity, studies have concluded that, regarding symptoms, individuals, usually with uncomplicated malaria, have developed an unusual situation in a disease characterised by weak or practically non-existent immunity [21]. During the last years (2010–2023), a total of 916 malaria cases were reported in the country, with a low mortality rate, with a total of nine deaths recorded (Table 1). Although relatively low, cases persisted, which may be linked to patients’ late care-seeking behaviours when faced with the first symptoms of the illness or inadequate case management. In turn, the mortality rate related to malaria did not exceed 0.006 (/1,000 inhabitants), the highest value in 2011, and the case fatality rate varied between 0.00 and 8.30 (/100 inhabitants) in the same year.

**Table 1:** Malaria cases, deaths, mortality, and case fatality rates in Cabo Verde from 2010 to 2023.

Year	Population	Total malaria cases	Number of deaths related to malaria	Mortality rate (/1000)	Case fatality rate (/100)
2010	477 859	47	1	0.002	2.1
2011	480 577	36	3	0.006	8.3
2012	482 285	36	1	0.002	2.8
2013	485 996	46	0	0.000	0.0
2014	488 719	46	1	0.002	2.2
2015	491 436	27	0	0.000	0.0
2016	493 465	75	1	0.002	1.3
2017	495 522	446	2	0.004	0.4
2018	497 558	21	0	0.000	0.0
2019	499 608	40	0	0.000	0.0
2020	501 657	10	0	0.000	0.0
2021	504 125	21	0	0.000	0.0
2022	506 595	28	0	0.000	0.0
2023	509 078	37	1	0.002	2.7
**Total**		**916**	**9**	**–**	**–**

## Discussion

### Cabo Verde as a malaria-free country: The main challenges, perspectives and the ahead

Understanding malaria patterns in countries where it spreads is crucial for creating effective control strategies [22]. A renewed global commitment to eradicating malaria is highlighted by the World Health Organization’s Global Technical Strategy for Malaria 2016–2030 [22–24]. This goal is also part of the Roll Back Malaria plan and aligns with the Sustainable Development Goals [25], which address major health issues, including malaria. Many countries aim to eliminate malaria [26,27], with at least 35 out of 91 countries with ongoing transmission expected to reduce cases by 90% by 2030 [28]. However, this requires strong political support at all levels [24,29,30].

In Cabo Verde, malaria elimination efforts focused on developing policies for low-permission areas, where the positivity rate is below 5% among febrile cases [31,32]. Interventions must be enhanced to prevent malaria reintroduction in an archipelago country that may limit the spread of malaria, but increased travel between islands due to economic growth presents challenges. Current measures, like spraying planes and boats in line with international health regulations, have limited impact compared to other countries in the elimination phase [22].

Historically, Cabo Verde saw three malaria-causing parasites: *P. falciparum, P. vivax*, and *P. malariae*. In the 1960s, *P. vivax* was more common than *P. falciparum*, but since 1994, *P. falciparum* has been responsible for all reported malaria cases, both native and imported. Most imported cases come from West African countries like Guinea Bissau and Angola. With increased travel to Africa and the Americas, Cabo Verde faces a constant risk of imported malaria. After years of absence, *P. vivax* reappeared in 2017 from Brazil and, with improved diagnostic methods, mixed infections, such as P. *falciparum* with *P. ovale* in 2022 and with *P. malariae* in 2023 were detected in the country [20]. As part of the Economic Community of West African States (ECOWAS) and with open borders, Cabo Verde has a risk of malaria reintroduction due to tourism and travel [13,33,34]. Therefore, ongoing investment is key to maintaining diagnostic capabilities. The biggest challenge in keeping malaria control gains is ensuring the financial resources to support these efforts [35]. The Global Fund to Fight AIDS, Tuberculosis, and Malaria (GFATM) has been a significant funding source since 2010, investing around 4.5 million Euros in malaria control efforts in Cabo Verde, focusing on vector control, case management, and program management [36]. Despite these achievements, GFATM has announced that its upcoming grant cycle from 2024 to 2026 will be the last for malaria. This means that the country’s government needs to find new partners and financing strategies, including involving private and international partners [37–40]. The WHO and other essential partners’ support will continue to play a crucial role in the overall elimination and certification process.

Recent analysis highlights gaps in human resources, training, and capacity building for malaria combat in Cabo Verde. This means effective engagement and strategies are needed, particularly in governance and leadership coordination [41]. Despite the technical support provided by the partners, key areas like entomology, epidemiology, and data management require special attention. The country’s case-based malaria surveillance system faces challenges, especially in managing imported cases and monitoring trends [42–44]. To sustain malaria elimination, it’s crucial to identify residual transmission foci and ensure timely treatment of asymptomatic and symptomatic infections [45–48]. While RDTs have improved access, they do not detect low-level infections, necessitating more sensitive tools for accurate diagnosis [49–51]. Although primaquine is used for treatment, G6PD deficiency poses risks, and the prevalence in the population necessitates enzyme testing for better management [52,53]. Despite no reported resistance to antimalarial treatments in Cabo Verde, monitoring for drug resistance is essential, considering the reality in Southeast Asian countries and its spread to Africa [54–57].

### Malaria data and the lessons learned

Cabo Verde has eliminated malaria, but it still faces challenges. Historically, the country has controlled malaria transmission twice, achieving WHO certification and becoming the fourth African country to achieve this goal [3]. However, a 2017 outbreak with 423 local cases [34] highlighted weaknesses in disease control, and the management of imported cases requires considerable adaptation to prevent reintroduction [58,59]. Since then, there have been no local cases, but imported cases continue to be reported, particularly from other Portuguese-speaking African countries [13,33,34].

The mobility of malaria-infected individuals poses challenges after the elimination, and in the actual situation of a free malaria country, preventing the reintroduction, spreading parasite drug resistance, straining country-to-country collaboration, and making routine data collection difficult, especially in resource-poor settings [60]. The cross-border mobility of malaria cases poses an obstacle in many countries [61], which is not problematic for the archipelagic country. While Cabo Verde’s geography as an archipelago mitigates some risks, globalization and tourism pose ongoing challenges. Despite a functional surveillance system, imported malaria remains complex, accounting for 23% of cases reported across 16 municipalities and eight inhabited islands [62–65]. These cases would undoubtedly be linked to these islands’ ecological and environmental situation [63], the greater circulation of the parasite and difficulties in control and prevention. On the remaining islands, few cases are registered, and the absence of local cases may be linked to environmental and climatic situations, with less movement of people from endemic countries. To effectively manage imported malaria cases, Cabo Verde must target geographic sources of infection, particularly in Lusophone countries like Angola, Guinea Bissau, and Senegal.

### Malaria vectors control in Cabo Verde: The translation and scale-up of novel strategies to the local level

Eleven mosquito species have been identified in Cabo Verde, with five significant vectors of diseases: *An. gambiae s.l., Aedes aegypti, Culex quinquefasciatus, Cx. pipiens ss,* and *Cx. Perexiguus* [66,67].

In sub-Saharan Africa, *An. gambiae* complex is the primary malaria vector. The species is widely distributed in the region, occupying more than 70% of the land, with polymorphic biting and resting behaviors depending on host availability and location inside or outside homes [68]. In Cabo Verde, *An. arabiensis* is the only known malaria vector [9,66,67,69–72] identified in the islands, with anthropophilic and marked exophilic behaviors [70–72], possibly influenced by indoor residual spraying (IRS) campaigns that have been implemented over the years [72,73]. Nonetheless, its distribution and more ecological data must be studied and confirmed. A recent study was carried out to update the distribution data of the natural populations of *An. arabiensis,* confirmed the presence of the species in the Santiago (municipalities of Praia, Tarrafal, São Salvador do Mundo, Santa Cruz, São Lourenço dos Órgãos, São Miguel, and Santa Catarina), São Vicente, Boavista and Maio islands [74].

A multi-sector approach is necessary to combat vector-borne diseases, emphasizing environmental health and political commitment to effective long-term vector control [45,75–77]. Historically, vector control has been crucial in malaria eradication efforts in Cabo Verde, with previous IRS campaigns successfully interrupting transmission [17,68]. The Integrated Vector Control Manual advocates an integrated approach to tackle multiple diseases, focusing on larval and adult control methods and community engagement [78], and an integration approach for all different species of mosquito vectors, mainly *Aedes aegypti*, responsible for dengue and Zika epidemics in the country [67,79]. Long-lasting insecticidal nets (LLINs) are not widely used for prevention; they are mainly distributed during malaria epidemics in hospitals. Although DDT was effective in the past for malaria control [80], the WHO now recommends less harmful insecticides like pyrethroids and organophosphates, which are currently in use.

Among the three classes of authorised insecticides, only the first two are used in the fight against malaria in Cabo Verde; deltamethrin is 0.5% for IRS activities in adults and temephos (abate) for larval control [81]. In addition to chemicals, the country recommends using larvivorous fish and petroleum derivatives, which are limited to specific areas. With international health regulations, the government adopted the use of α-permethrin in spraying aircraft. Spraying campaigns are organised in two annual cycles nationwide, usually before and after rains, emphasising the most affected areas, namely Praia and Boavista.

Research on mosquito behavior and insecticide sensitivity is crucial for controlling malaria in Cabo Verde. Studies from 2011 indicated that *An. arabiensis* from Santiago Island was generally sensitive to several insecticides, including deltamethrin and alpha-cyhalothrin [81]. However, variations in susceptibility to temephos were observed, with mortality rates ranging from 43.1% to 90.9% [82]. Recent findings show mutations linked to resistance, emphasizing the need to regularly monitor insecticide resistance at least once a year to ensure effective vector control [83,84]. Given *An. arabiensis’s* behavioral polymorphism and the lack of comprehensive entomological data in the country, more research is needed on its distribution, behavior, and ecology. This includes studying the Entomological Inoculation Rate (EIR) and environmental conditions to develop effective control strategies. Integrated vector control is recommended to minimize resistance, though its universal application has limitations. Current methods, such as IRS and LLIN, may not effectively target outdoor populations of mosquitoes [85–87], so innovative strategies, including molecular tools and genetic approaches like gene drive technology, offer promising avenues for vector control in Cabo Verde’s unique archipelagic environment [88,89]. Strengthening local biological control, monitoring, and infrastructure is essential for a coordinated response.

### Climatic changes, new vector ecological habits and behaviour change

Climate change impacts all regions, leading to extreme weather and threatening Sustainable Development Goals, especially in vulnerable communities like Cabo Verde. The archipelago’s insularity and climatic characteristics exacerbate the effects of climate change on ecosystems and the population. Cabo Verde faces a “climate shock,” with projections indicating hotter and drier conditions [90]. The IPCC models suggest average temperatures could rise by up to 2.5 °C and humidity and precipitation may decrease by 5–10% annually, with sea level rise expected between 0.13 and 1.4 m by century’s end [91]. Research shows health risks from climate change, including temperature-related illnesses and vector-borne diseases [92–96]. Malaria, a climate-sensitive disease in African countries, including Cabo Verde, is mainly linked to temperature and rainfall [97–100]. The islands have a dry Sahelian climate, with minimal rain, making malaria transmission less favorable. Previous studies focused on Santiago and Boavista, revealing correlations between malaria cases and climatic factors. Still, there’s a lack of data on Brava and Santo Antão, which have not experienced indigenous malaria cases [33]. Agricultural areas in Santiago, Santa Catarina, and Santa Cruz reported malaria cases, possibly related to increased vector density from breeding sites.

Recently, studies have demonstrated specific shifts in vector behaviour following the introduction of malaria vector control interventions [101–103], including species shifts, shifts toward early evening and early morning biting, outdoor resting and biting, and zoophiles. To be more resilient to the impact of climate change on health, especially the rising temperatures and malaria, Cabo Verde must strengthen its entomological surveillance capacity [100,104–109].

Another main concern for Cabo Verde is the potential introduction and establishment of new malaria vectors. The species, *An. stephensi*, historically considered an Asian malaria vector, was detected in Africa in Djibouti, Ethiopia, Somalia, Sudan, Ghana, Kenya, and Nigeria [110]. Given these results and the *An. stephensi*’s ability to invade the new area, Cabo Verde should not rule out its introduction, as well as other species, which could be other competent vectors.

### A robust and functional monitoring system will maintain the malaria detection capacity at all levels

Monitoring and evaluation (M&E) systematically track public health programs over time, focusing on malaria program scale-up primarily on burden reduction, specifically morbidity and mortality [111]. For countries in the elimination stage, measuring malaria morbidity and mortality has become challenging due to a high proportion of asymptomatic cases [112]. Therefore, M&E must shift to detecting malaria infections and understanding transmission dynamics.

Elimination monitoring integrates health data, population census, and needs assessment to enable personalized, rapid interventions [104]. Effective surveillance should utilize tools like GIS and SDSS to predict infection risk and guide local responses [42,108,113]. Mapping vulnerability risk and developing strategies for managing imported cases remain significant challenges. In Cabo Verde, initial mapping efforts began during the 2017 epidemic, yet comprehensive vector distribution studies are still needed for an effective malaria response [33].

Field research during the 2017 outbreaks suggests that receptivity can sometimes be high in transmission foci, regardless of ecological conditions. The dynamics of transmission in an active focus studied in Santa Catarina de Santiago in 1995 [114] showed that 41% of the inhabitants were affected by the same lineage of clonal parasites and by all age groups affected. According to this study, the parasite can maintain asymptomatic infections despite long drought and non-transmission periods. Despite residual local cases in Santiago and recently in Boavista, whose outbreaks are well known, it was not until 2017 that mapping of breeding sites and reported cases began in Praia.

Cabo Verde must strengthen its surveillance system to assess trends and respond effectively, especially as a malaria-free country. Research is necessary to improve testing capacity for low parasitaemia detection and evaluate the efficacy of interventions like IRS and larval control. New technologies for mapping and surveillance, along with the systematic review of past elimination efforts, are crucial for sustaining progress and implementing effective malaria prevention strategies [115,116].

### Epidemiological surveillance, operational research and quality of interventions

Malaria epidemiological surveillance in Cabo Verde is conducted through structured procedures that provide information on cases and deaths, essential for program planning and evaluation [77,117]. The National Directorate of Health, along with the NMCP and the SVIRE, coordinates efforts to monitor malaria prevalence and incidence and promote health research. The Integrated Disease Surveillance and Response (VID-R) strategy, implemented in 2002 and revised in 2007, involves systematic investigation of malaria cases by health delegations and ensures appropriate responses through the availability of RDT [118].

The National Institute of Public Health (INSP) leads malaria research in Cabo Verde, bolstered by the 2017 establishment of a medical entomology laboratory. This research includes projects like Target Malaria, WADE, and Freedom from Infection, focusing on malaria prevalence, environmental impacts, vector behavior, and more [13,34,119–121]. Collaborations with national and international institutions, such as the University of Cabo Verde (Uni-CV), Jean Piaget University, Pasteur Institute of Dakar, the Institute of Hygiene and Tropical Medicine (IHMT), and others, enhance research quality.

Cabo Verde’s functional surveillance system faces challenges in improving its sustainability for malaria control [108]. As the WHO Expert Committee highlights, effective surveillance is essential for disease prevention and elimination [122,123]. The country has implemented strategic passive and active case detection methods to manage reduced malaria cases [24,30,113]. Also, the WHO’s Framework for Elimination advises a shift towards systems that enable rapid and detailed case reporting [30]; however, Cabo Verde struggles with local alert systems, data integration, and analytic improvements. The WHO’s Global Technical Strategy emphasizes the need for integrated health data to support targeted interventions. For Cabo Verde, surveillance and monitoring require integrating health data, population census, information on the acquisition of needs and others, supporting actions and forecasting programmes to obtain personalised results that would lead to rapid and targeted interventions [111,124]. This system allows the early detection of malaria cases with the investigation, notification and response in real-time following national malaria surveillance procedures, including the management of imported cases, particularly at maritime and continental borders. Ideal surveillance systems should use tools such as GIS and SDSS for georeferencing and predicting infection risk. Additionally, this information should be used to direct and guide effective and targeted responses at the local level. In elimination and reintroduction prevention programs in malaria eradication, mapping vulnerability risk is crucial for surveillance operations. Specific strategies for imported cases remain significant challenges for the country. The country also needs to re-evaluate the service integration delivery for malaria [107], reinforce the communications for the governance of malaria programs [26], suppress the human resources and capacity building needs [38] and intensive inter-country cooperation [124] and communication [125], as key strategies to prevent malaria reintroduction. Investment in studies and research must be reinforced in the country, creating conditions for various questions, challenges, and situations to be answered and clarified. Thus, providing evidence for making the best decisions can contribute to Cabo Verde’s perpetuity and guarantee the sustainability of a malaria-free country, a reference for Africa and the world.

## Conclusion

With a long history of malaria cases in the country, Cabo Verde was the first sub-Saharan African country to achieve malaria elimination. With the reduction of the last local cases in two islands, Santiago in 2017 and Boavista in 2015, the certification was achieved on January 12th, 2024, by the WHO. With this certification, Cabo Verde joins the group of 43 countries and one territory the WHO has granted as malaria-free, being the fourth country in Africa, joining Mauritius, Morocco, and Algeria, certified in 1973, 2010, and 2019, respectively.

Certification of malaria elimination will drive positive economic development in many sectors of Cabo Verde. The systems and structures created to eliminate malaria have strengthened the health system and will be used to combat other mosquito-borne diseases. The goal was achieved, and with it, challenges will be faced for the country to perpetuate this achievement and maintain its sustainability, focusing on preventing reintroduction. Investments are necessary to overcome the various tasks in assembling a robust and functional entomological and epidemiological surveillance system to respond to imported cases with immediate detection, correct treatment, and follow-up. Leadership in program coordination and management, training human resources in various areas, investment in research and investigation, and implementation of an effective anti-vector control system are the challenges that the country needs to surmount to guarantee the sustainability of this success.

## Supporting information

S1 Table The main activities implemented in the malaria history of malaria control in Cabo Verde. From the first moments of the fight against the disease in 1953 to eliminating and certifying the country as malaria-free in 2024.
(PDF)

S2 Table Number of malaria cases reported in Cape Verde, by island and municipality, according to the origin of the case, in the period 1988–2022. (XLSX)
