# Supplementary material for: Situational analysis of malaria in Cabo Verde: From endemic control to elimination, history, cases data and challenges ahead
Source: PLOS Glob Public Health. 2025 Jan 10;5(1):e0004153. doi: 10.1371/journal.pgph.0004153 (PMC11723648; doi:10.1371/journal.pgph.0004153)
Supplement: S1 Table — From the first moments of the fight against the disease in 1953 to eliminating and certifying the country as malaria-free in 2024. (PDF) [file pgph.0004153.s001.docx]

**Supplementary Table 01:** The main activities implemented in the malaria history of malaria control in Cabo Verde. From the first moments of the fight against the disease in 1953 to eliminating and certifying the country as malaria-free in 2024**.**

| **Date (Year)** | **Main Activitivities** |
| --- | --- |
| **1953** | The launch of the first malaria elimination campaign in Cabo Verde, which lasted seven years, targeted the most affected areas. Operations were intensified and expanded under the Global Malaria Eradication Program (Ribeiro et al., 1980). |
| **1955** | The creation of the prestigious Permanent Mission to Study and Combat Endemic Diseases in Cape Verde, which began to coordinate the prevention and control of malaria and other endemic diseases, having developed essential studies and initiated impactful measures to combat this disease and its vectors. According to Meira (1952 and 1959), *An. gambiae* was soon eradicated from the island of Sal. Cambournac (1971) reported that all mosquitoes were eradicated in Sal and S. Vicente. The same author pointed out that all mosquito species have been eradicated and eliminated on the island of Maio. Indoor residual spraying (IRS) with DDT was applied gradually in each isle until transmission was interrupted. Active case-finding and larval control were also implemented, using petroleum in mosquito breeding sites and larvivorous fish (*Gambusia affinis*). Santiago, the largest and most affected island, had full IRS campaigns twice yearly for four years (until 1967). |
| **1967** | Local malaria transmission was eliminated (up to and including 1972). The last autochthonous case throughout the archipelago was recorded on Santiago Island in 1967. |
| **1969** | The IRS campaigns were halted in 1969. Studies showed that the only malaria vector in the archipelago, *Anopheles arabiensis*, had been eliminated from all islands except Santiago. |
| **1973** | With the dismantling of malaria control services and the absence of a technical nucleus to ensure the follow-up of actions, the re-establishment of local transmission was inevitable. Thus, autochthonous malaria reappeared in Santiago, Santa Catarina and Santa Cruz municipalities, with 149 autochthonous cases reported. Preventive measures were taken in active hotspots in 1974 and 1975. After that, the number of cases decreased from 149 in 1973 to 20 autochthonous cases in 1976. |
| **1978** | A new outbreak occurred, with 844 cases and 13 deaths, peaking in 1978, mainly in Santa Catarina and Santa Cruz on the island of Santiago. The epidemic peak occurred in November, and a third of autochthonous cases were due to Plasmodium vivax. New IRS campaigns were carried out every six months for five years (1978 to 1982). |
| **1978** | The Malaria Fight Brigade was created to respond to the void left by dismantling malaria control services in 1969, the resumption of local transmission of the disease (1973) and the epidemic that began in 1977. |
| **1989** | Creation of the NMCP, replacing the Malaria Fight Brigade and the previously existing vector control team. Until 2009, several action plans were developed and implemented but did not focus on malaria elimination. Maintenance of the number of malaria cases at a low level with, however, three outbreaks of around 100 cases, located in separate locations on the island of Santiago, in 1995 (Achada Leite – Sta Catarina), in 2000 (Assomada – Sta Catarina) and 2001 (Achada Fátima and Salina – Sta Cruz); then, small outbreaks of transmission appeared on the island of Boavista, considered non-endemic since the end of the 1960s. In 2006 was a critical year with the increased malaria burden in Santiago and the extension of the risk zone to Boavista. Between 1990 and 2009, 1293 cases were reported, including 929 classifieds as indigenous (all due to *P. falciparum*) and the remaining cases imported. |
| **2009** | Preparation of the first National Strategic Plan for the Pre-elimination of malaria for 2009-2013, whose objective was to reduce the incidence to less than one case / 1.000 inhabitants. |
| **2010 - 2012** | Malaria endemicity has decreased, significantly reducing the annual number of autochthonous cases to a single case reported in 2012. |
| **2014** | That year (2014), the island of Boavista reported one Indigenous case after decades of no cases. |
| **2015** | Autochthonous transmission on the island of Boavista continued in 2015, with the notification of 4 autochthonous cases, which motivated the carrying out of localised IRS operations for two consecutive years |
| **2016** | Commitment to the WHO E2020 initiative, which involves 21 countries worldwide, can potentially eliminate malaria by 2020. |
| **2017** | In July 2017, a new malaria epidemic occurred in Praia, with 446 cases in total, 423 local cases, and an unusual epidemic peak. |
| **2018 – 2020** | The elimination of local malaria transmission for the third time, with the notification of zero indigenous cases from February 2018 to June 2023. During these periods, a Program Review was done, a new Strategic plan for malaria elimination, 2020-2024; and New Standard Operating Procedures for different subjects were also elaborated and adopted to achieve malaria elimination |
